# Factors Associated with Participation in Elementary School–Based SARS-CoV-2 Testing — Salt Lake County, Utah, December 2020–January 2021

**DOI:** 10.15585/mmwr.mm7015e1

**Published:** 2021-04-16

**Authors:** Nathaniel M. Lewis, Rebecca B. Hershow, Victoria T. Chu, Karen Wu, Alison T. Milne, Nathan LaCross, Mary Hill, Ilene Risk, Adam L. Hersh, Hannah L. Kirking, Jacqueline E. Tate, Snigdha Vallabhaneni, Angela C. Dunn

**Affiliations:** ^1^CDC COVID-19 Response Team; ^2^Epidemic Intelligence Service, CDC; ^3^Utah Department of Health; ^4^Granite School District, Utah; ^5^Salt Lake County Health Department, Salt Lake City, Utah; ^6^Department of Pediatrics, Division of Infectious Diseases, University of Utah, Salt Lake City, Utah.

During December 3, 2020–January 31, 2021, CDC, in collaboration with the University of Utah Health and Economic Recovery Outreach Project,* Utah Department of Health (UDOH), Salt Lake County Health Department, and one Salt Lake county school district, offered free, in-school, real-time reverse transcription–polymerase chain reaction (RT-PCR) saliva testing as part of a transmission investigation of SARS-CoV-2, the virus that causes COVID-19, in elementary school settings. School contacts^†^ of persons with laboratory-confirmed SARS-CoV-2 infection, including close contacts, were eligible to participate (*1*). Investigators approached parents or guardians of student contacts by telephone, and during January, using school phone lines to offer in-school specimen collection; the testing procedures were explained in the preferred language of the parent or guardian. Consent for participants was obtained via an electronic form sent by e-mail. Analyses examined participation (i.e., completing in-school specimen collection for SARS-CoV-2 testing) in relation to factors^§^ that were programmatically important or could influence likelihood of SARS-CoV-2 testing, including race, ethnicity, and SARS-CoV-2 incidence in the community (*2*). Crude prevalence ratios (PRs) were calculated using univariate log-binomial regression.^¶^ This activity was reviewed by CDC and was conducted consistent with federal law and CDC policy.**

Among 856 unique student contacts at 20 elementary schools, 594 who were exposed to 33 index patients at 13 elementary schools were analyzed (Table).^††^ Among 594 student contacts, 438 (74%) participated (range = 59%–82% across schools), parents or guardians of 100 (17%) students refused, and 56 (9%) could not be reached (Table). Student testing outside of the investigation was not evaluated. Among 436 participants with available information,^§§^ parents or guardians of 230 (53%) consented to participation after the first contact attempt, an additional 134 (31%) after two attempts, and a further 72 (17%) after three attempts.

**TABLE:** Characteristics associated with participation in school SARS-CoV-2 testing among student contacts (N = 594) — 13 elementary schools, Salt Lake County, Utah, December 2020–January 2021*

Characteristic	No. (%)		Prevalence ratio (95% CI)
	Total	Participants	
	(N = 594)	(n = 438)	
**Student characteristic**			
**Race/Ethnicity**			
White, non-Hispanic	**285**	190 (66.7)	Ref
White, Hispanic/Latino	**213**	172 (80.8)	1.21 (1.09–1.35)
Racial minority^†^	**96**	76 (79.2)	1.19 (1.04–1.35)
**Grade in school**			
Kindergarten–grade 2	**258**	197 (76.4)	Ref
Grades 3–4	**162**	118 (72.8)	0.95 (0.85–1.07)
Grades 5–6^§^	**174**	123 (70.7)	0.93 (0.82–1.04)
**Identified as a close contact to index patient^¶^**			
No	**428**	314 (73.4)	Ref
Yes	**166**	124 (74.7)	1.02 (0.92–1.13)
**Family member (including nonhousehold) ever received positive SARS-CoV-2 test result**			
No	**534**	389 (72.8)	Ref
Yes	**60**	49 (81.7)	1.12 (0.98–1.28)
**School/Investigation characteristic**			
**Cumulative SARS-CoV-2 incidence rate by school**			
≤51 cases per 1,000 persons**	**305**	216 (70.8)	Ref
>51 cases per 1,000 persons	**289**	222 (76.8)	1.08 (0.99–1.19)
**No. of school cases during 14 days before testing date**			
1–4	**402**	300 (74.6)	Ref
>4	**192**	138 (71.9)	0.96 (0.87–1.07)
**Days from last school exposure to first time contacted^††^**			
2–4	**142**	106 (74.6)	Ref
5–7	**331**	245 (74.0)	0.99 (0.88–1.11)
8–12	**118**	85 (72.0)	0.97 (0.83–1.12)
**Days from last school exposure to test date**			
6–7	**316**	231 (73.1)	Ref
8–10	**278**	207 (74.5)	1.02 (0.93–1.12)
**Month of testing**			
December	**227**	156 (68.7)	Ref
January	**367**	282 (76.8)	1.12 (1.01–1.24)
**ZIP code–level characteristic^††^**			
**Cumulative SARS-CoV-2 incidence rate by ZIP code since March 2020**			
≤11,461 cases per 100,000 persons^§§^	**301**	210 (69.8)	Ref
>11,461 cases per 100,000 persons	**292**	228 (78.1)	1.12 (1.02–1.23)
**Deprivation level^¶¶^**			
Very low to average	**315**	226 (71.7)	Ref
High to very high	**278**	212 (76.3)	1.06 (0.97–1.17)
**Masking compliance rate by ZIP code since May 2020**			
≥81.6%***	**299**	230 (76.9)	Ref
<81.6%	**294**	208 (70.7)	0.92 (0.84–1.01)

**Abbreviations:** CI = confidence interval; Ref = Reference group.

* Log-binomial regression was conducted to calculate crude prevalence ratios and 95% CIs to identify correlates of participation. Prevalence ratio estimates that did not include 1.0 were considered statistically significant at p<0.05. Participation was defined as completing in-school specimen collection for reverse transcription–polymerase chain reaction SARS-CoV-2 testing.

^†^ Includes non-Hispanic and Hispanic Asian, non-Hispanic and Hispanic Black/African American, non-Hispanic Native Hawaiian or Other Pacific Islander, non-Hispanic and Hispanic American Indian or Alaska Native, and non-Hispanic and Hispanic Multiracial. Among 96 students who identified as members of a racial minority group, 11 (11%) also identified as Hispanic or Latino.

^§^ All students in this category were in grades 5 or 6 except for two students who were in grade 7 or higher because they were identified as school contacts of the same index patient.

^¶^ Close school contacts were defined as persons within 6 ft of the index patient for a cumulative total of ≥15 minutes over a 24-hour period.

** Median SARS-CoV-2 incidence rate across schools included in the analysis.

^††^ Missing data: ZIP code was missing for one nonparticipating student; days between last exposure date and first time family was contacted was missing for three students.

^§§^ Median SARS-CoV-2 incidence rate across students’ ZIP codes included in the analysis.

^¶¶^ This is a composite index calculated using nine indicators from the Utah Behavioral Risk Factor Surveillance System: 1) median family income; 2) income disparity (a logarithmic ratio of households with <$10,000 income to ≥$50,000 income); 3) percentage of home ownership; 4) percentage of unemployment; 5) percentage of families below poverty threshold; 6) percentage of single-parent households with children aged <18 years; 7) percentage of population aged ≥25 years with <9 years of education; 8) percentage of population aged ≥25 years with at least a high school diploma; and 9) percentage of population at <150% of the poverty threshold. The index is divided into quintiles (very low, low, average, high, and very high).

*** Median masking compliance rate among residential ZIP codes of students included in the analysis.

Compared with non-Hispanic White students, participation in the testing program was higher among students identifying as Hispanic/Latino White (PR = 1.21) and among members of a racial minority group^¶¶^ (PR = 1.19). Participation was higher in January (PR = 1.12) than in December. Compared with students living in ZIP codes with lower SARS-CoV-2 incidence than the median in all residential ZIP codes of students included in the analysis (11,461 cases per 100,000 persons), participation was higher among those living in ZIP codes with incidences higher than the median (PR = 1.12). No differences were found based on grade level, close contact with the index patient, having a family member ever receive a positive SARS-CoV-2 test result, cumulative school incidence, number of recent school cases, number of days from exposure to first contact or to testing, ZIP code–level deprivation score (a composite measure of socioeconomic disadvantage) (*3*), or ZIP code–level mask compliance, estimated as the percentage of adult residents who reported always wearing a mask in public.

In Utah’s socioeconomically disadvantaged areas, which have large proportions of uninsured and racial and ethnic minority residents, SARS-CoV-2 incidence is elevated, but testing rates are similar to those in other areas; this discrepancy could reflect a lack of access to testing (*2*). The sociodemographic differences in participation rates observed in this investigation could also suggest a higher level of concern about COVID-19 school safety among racial and ethnic minority parents (*4*) or less concern or better access to other testing resources among non-Hispanic White households. In-school specimen collection could therefore be a useful strategy for facilitating SARS-CoV-2 testing among those at higher risk for infection, who might also have limited access to testing. Higher participation in January compared with December could reflect the absence of potential holiday disincentives to testing or the investigation team’s use of school phone lines for recruitment in January.

As schools consider reopening, in-school specimen collection for SARS-CoV-2 testing could help reach potentially underserved populations to reduce community transmission (*5*,*6*). Explaining testing procedures in a parent’s or guardian’s preferred language, as was done in this situation, might also be important for promoting participation. One limitation is that testing was conducted among persons with a known SARS-CoV-2 exposure; in-school specimen collection without known exposures might result in different participation rates. A second limitation is that testing history among participants was not known; therefore, the degree to which access to testing in the community influenced participation is unknown. However, the high participation rate for RT-PCR saliva testing suggests potential scalability to other school testing strategies, including screening testing (*7*,*8*). School districts should continue universal mask use, physically distancing ≥3 ft (or as much as possible), and quarantining close contacts of persons with COVID-19 (*8*).

## Footnotes

*
https://eccles.utah.edu/utah-hero/

† To detect any potential school-associated transmission beyond close contacts of cases and to assess broader acceptability of in-school specimen collection school contacts were defined in this investigation as students or staff members in contact with the index patient for a cumulative total of ≥15 minutes during a 24-hour period in a classroom, cafeteria, school bus, or recess space during the index patient’s infectious period. Close school contacts were defined as persons within 6 ft of the index patient for a cumulative total of ≥15 minutes over a 24-hour period. Infectious period was estimated as 2 days before to 10 days after symptom onset (if symptomatic) or first positive specimen collection date (if asymptomatic). https://www.cdc.gov/coronavirus/2019-ncov/php/contact-tracing/contact-tracing-plan/investigating-covid-19-case.html

§ Student demographics and school-level characteristics were obtained from the school district. Incidence by each student’s ZIP code of residence was obtained from UDOH. ZIP code–level deprivation and mask compliance, estimated as the percentage of the adult population reporting that they always wear a mask in public settings, were obtained from the Utah Behavioral Risk Factor Surveillance System. https://ibis.health.utah.gov/ibisph-view/query/selection/brfss/BRFSSSelection.html

¶ Prevalence ratio estimates that did not include 1.0 were considered statistically significant at p<0.05.

** 45 C.F.R. part 46, 21 C.F.R. part 56; 42 U.S.C. Sect. 241(d); 5 U.S.C. Sect. 552a; 44 U.S.C. Sect. 3501 et seq.

†† Students were excluded if testing was offered on days off or during online learning days, potentially requiring additional transportation (253), or if they attended a private school (nine).

§§ The number of times the family was contacted was missing for two participating students.

¶¶ Includes non-Hispanic and Hispanic Asian, non-Hispanic and Hispanic Black/African American, non-Hispanic Native Hawaiian or Other Pacific Islander, non-Hispanic and Hispanic American Indian or Alaska Native, and non-Hispanic and Hispanic Multiracial. Among 96 students that identified as being from a racial minority group, 11 (11%) also identified as Hispanic or Latino.
